# Crystal structure of 4-phenyl-1-{2-[(2,4,6-tri­methyl­phen­yl)selan­yl]phen­yl}-1*H*-1,2,3-triazole

**DOI:** 10.1107/S2056989015003229

**Published:** 2015-02-25

**Authors:** Leandro R. S. Camargo, Julio Zukerman-Schpector, Anna M. Deobald, Antonio L. Braga, Edward R. T. Tiekink

**Affiliations:** a Departmento de Química, Universidade Federal de São Carlos, 13565-905 São Carlos, SP, Brazil; b Departmento de Química, Universidade Federal de Santa Maria, 97105-900 Santa Maria, RS, Brazil; c Departmento de Química, Universidade Federal de Santa Catarina, 88040-900 Florianópolis, SC, Brazil; d Department of Chemistry, University of Malaya, 50603 Kuala Lumpur, Malaysia

**Keywords:** crystal structure, organoselenium, hydrogen bonding, Se⋯N halogen bonding, C—H⋯π inter­actions

## Abstract

In the title compound, C_23_H_21_N_3_Se, the C-bound phenyl ring is almost coplanar with the central five-membered ring [dihedral angle = 2.84 (10)°], but the N-bound benzene ring is inclined [dihedral angle = 47.52 (10)°]. The dihedral angle between the Se-bound rings is 69.24 (9)°. An intra­molecular Se⋯N inter­action of 3.0248 (15) Å is noted. In the crystal, C—H⋯π inter­actions connect mol­ecules into double layers that stack along the *a* axis with no directional inter­actions between them.

## Related literature   

For background and synthesis of aryl­seleno-1,2,3-triazoles, including of the title compound, see: Deobald *et al.* (2011 ▸). For Se⋯N inter­actions, see: Pati & Zade (2014 ▸). For a related organoselenium compound with a 1,2,3-triazole residue, see: Camargo *et al.* (2015 ▸).
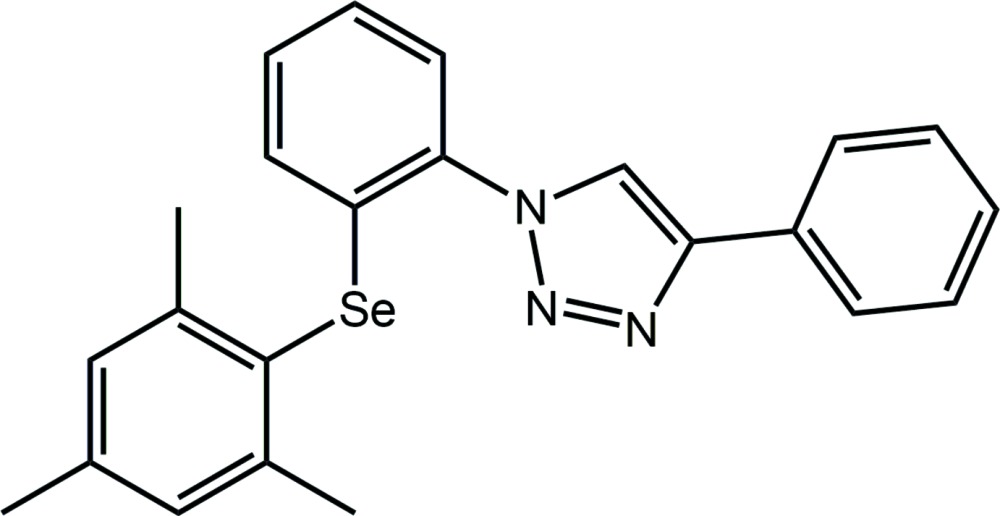


## Experimental   

### Crystal data  

C_23_H_21_N_3_Se
*M* *_r_* = 418.39
Monoclinic, 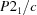

*a* = 21.3924 (4) Å
*b* = 6.9332 (1) Å
*c* = 12.9204 (2) Å
β = 92.231 (2)°
*V* = 1914.87 (5) Å^3^
*Z* = 4
Mo *K*α radiation
μ = 1.97 mm^−1^
*T* = 100 K
0.20 × 0.15 × 0.10 mm

### Data collection  

Agilent SuperNova CCD diffractometer
Absorption correction: multi-scan (*CrysAlis PRO*; Agilent, 2011 ▸) *T*_min_ = 0.809, *T*_max_ = 1.000
9275 measured reflections
4252 independent reflections
3740 reflections with *I* > 2σ(*I*)
*R*_int_ = 0.026

### Refinement  

*R*[*F* ^2^ > 2σ(*F* ^2^)] = 0.030
*wR*(*F* ^2^) = 0.063
*S* = 1.03
4252 reflections
247 parameters
H-atom parameters constrained
Δρ_max_ = 0.38 e Å^−3^
Δρ_min_ = −0.39 e Å^−3^

### 

Data collection: *CrysAlis PRO* (Agilent, 2011 ▸); cell refinement: *CrysAlis PRO*; data reduction: *CrysAlis PRO*; program(s) used to solve structure: *SIR2014* (Burla *et al.*, 2015 ▸); program(s) used to refine structure: *SHELXL2014* (Sheldrick, 2015 ▸); molecular graphics: *ORTEP-3 for Windows* (Farrugia, 2012 ▸) and *DIAMOND* (Brandenburg, 2006 ▸); software used to prepare material for publication: *MarvinSketch* (ChemAxon, 2010 ▸) and *publCIF* (Westrip, 2010 ▸).

## Supplementary Material

Crystal structure: contains datablock(s) I, New_Global_Publ_Block. DOI: 10.1107/S2056989015003229/hg5433sup1.cif

Structure factors: contains datablock(s) I. DOI: 10.1107/S2056989015003229/hg5433Isup2.hkl

Supporting information file. DOI: 10.1107/S2056989015003229/hg5433Isup3.cml

**Fig. 1.**
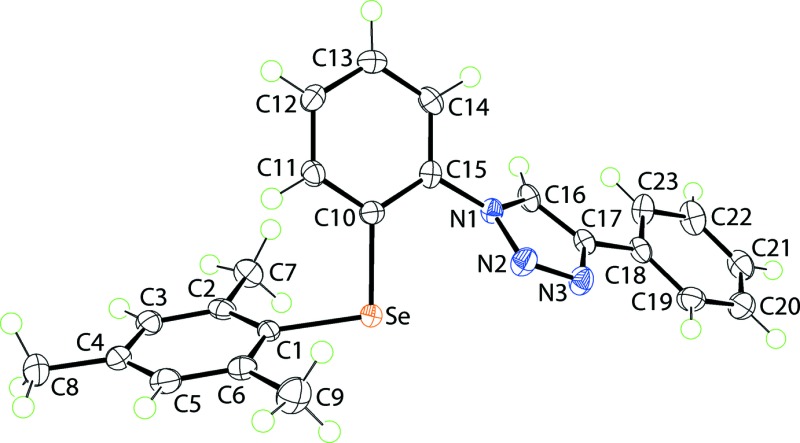
The molecular structure of the title compound showing the atom-labelling scheme and displacement ellipsoids at the 70% probability level.

**Fig. 2.**
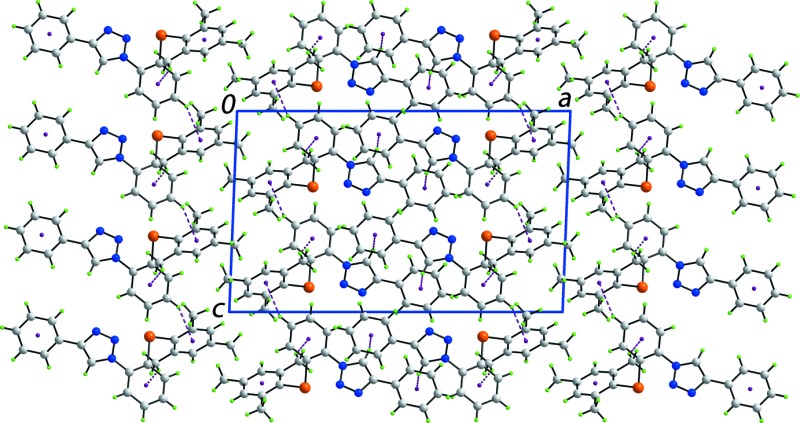
A view in projection down the b axis of the unit-cell contents. The C—H···π interactions are shown as purple dashed lines.

. DOI: 10.1107/S2056989015003229/hg5433fig1.tif
The mol­ecular structure of the title compound showing the atom-labelling scheme and displacement ellipsoids at the 70% probability level.

b . DOI: 10.1107/S2056989015003229/hg5433fig2.tif
A view in projection down the *b* axis of the unit-cell contents. The C—H⋯π inter­actions are shown as purple dashed lines.

CCDC reference: 1049547

Additional supporting information:  crystallographic information; 3D view; checkCIF report

## Figures and Tables

**Table 1:** Hydrogen-bond geometry (, ) *Cg*1, *Cg*2 and *Cg*3 are the centroids of the C1C6, C10C15 and C18C23 rings, respectively.

*D*H*A*	*D*H	H*A*	*D* *A*	*D*H*A*
C12H12*Cg*1^i^	0.95	2.68	3.481(2)	143
C7H7a*Cg*2^ii^	0.98	2.61	3.492(2)	150
C16H16*Cg*3^iii^	0.95	2.66	3.399(2)	135

Symmetry codes: (i) 
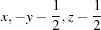
; (ii) 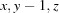; (iii) 
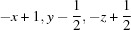
.

## supplementary crystallographic information

### S1. Experimental

The compound was prepared in accord with the literature (Deobald *et al.*, 2011). Crystals were obtained by taking 200 mg of sample into a sample vial containing methanol (5 ml) and ethyl acetate (5 ml) and letting it stand at room temperature.

### S2. Refinement

Carbon-bound H-atoms were placed in calculated positions (C—H = 0.95 to 0.99 Å) and were included in the refinement in the riding model approximation, with *U_iso_*(H) = 1.2–1.5*U_eq_*(C).

### Crystal data

**Table:**

C_23_H_21_N_3_Se	*F*(000) = 856
*M**_r_* = 418.39	*D*_x_ = 1.451 Mg m^−^^3^
Monoclinic, *P*2_1_/*c*	Mo *K*α radiation, λ = 0.71073 Å
*a* = 21.3924 (4) Å	Cell parameters from 4584 reflections
*b* = 6.9332 (1) Å	θ = 2.4–29.3°
*c* = 12.9204 (2) Å	µ = 1.97 mm^−^^1^
β = 92.231 (2)°	*T* = 100 K
*V* = 1914.87 (5) Å^3^	Prism, colourless
*Z* = 4	0.20 × 0.15 × 0.10 mm

### Data collection

**Table:**

Agilent SuperNova CCD diffractometer	3740 reflections with *I* > 2σ(*I*)
Radiation source: SuperNova (Cu) X-ray Source	*R*_int_ = 0.026
ω scans	θ_max_ = 27.5°, θ_min_ = 2.9°
Absorption correction: multi-scan (*CrysAlis PRO*; Agilent, 2011)	*h* = −25→27
*T*_min_ = 0.809, *T*_max_ = 1.000	*k* = −8→7
9275 measured reflections	*l* = −16→12
4252 independent reflections	

### Refinement

**Table:**

Refinement on *F*^2^	0 restraints
Least-squares matrix: full	Hydrogen site location: inferred from neighbouring sites
*R*[*F*^2^ > 2σ(*F*^2^)] = 0.030	H-atom parameters constrained
*wR*(*F*^2^) = 0.063	*w* = 1/[σ^2^(*F*_o_^2^) + (0.0204*P*)^2^ + 1.1744*P*] where *P* = (*F*_o_^2^ + 2*F*_c_^2^)/3
*S* = 1.03	(Δ/σ)_max_ = 0.001
4252 reflections	Δρ_max_ = 0.38 e Å^−^^3^
247 parameters	Δρ_min_ = −0.39 e Å^−^^3^

### Special details

**Table:**

Geometry. All e.s.d.'s (except the e.s.d. in the dihedral angle between two l.s. planes) are estimated using the full covariance matrix. The cell e.s.d.'s are taken into account individually in the estimation of e.s.d.'s in distances, angles and torsion angles; correlations between e.s.d.'s in cell parameters are only used when they are defined by crystal symmetry. An approximate (isotropic) treatment of cell e.s.d.'s is used for estimating e.s.d.'s involving l.s. planes.

### Fractional atomic coordinates and isotropic or equivalent isotropic displacement parameters (Å^2^)

**Table:**

	*x*	*y*	*z*	*U*_iso_*/*U*_eq_	
Se	0.76393 (2)	0.40426 (3)	0.12277 (2)	0.01683 (7)	
N1	0.65328 (7)	0.6076 (2)	0.23160 (12)	0.0130 (3)	
N2	0.65949 (7)	0.6956 (2)	0.13866 (12)	0.0175 (4)	
N3	0.60302 (7)	0.7185 (2)	0.09728 (12)	0.0171 (3)	
C1	0.83935 (8)	0.2521 (3)	0.13795 (14)	0.0132 (4)	
C2	0.84041 (9)	0.0834 (3)	0.19790 (14)	0.0143 (4)	
C3	0.89540 (9)	−0.0249 (3)	0.20417 (15)	0.0168 (4)	
H3	0.8966	−0.1393	0.2447	0.020*	
C4	0.94859 (9)	0.0300 (3)	0.15272 (15)	0.0181 (4)	
C5	0.94552 (9)	0.1966 (3)	0.09317 (15)	0.0182 (4)	
H5	0.9814	0.2347	0.0571	0.022*	
C6	0.89188 (9)	0.3100 (3)	0.08433 (14)	0.0157 (4)	
C7	0.78440 (9)	0.0169 (3)	0.25528 (16)	0.0192 (4)	
H7A	0.7944	−0.1043	0.2912	0.029*	
H7B	0.7490	−0.0033	0.2060	0.029*	
H7C	0.7734	0.1152	0.3060	0.029*	
C8	1.00802 (10)	−0.0852 (4)	0.16441 (17)	0.0291 (5)	
H8A	0.9984	−0.2228	0.1570	0.044*	
H8B	1.0277	−0.0615	0.2330	0.044*	
H8C	1.0367	−0.0464	0.1109	0.044*	
C9	0.89247 (10)	0.4894 (3)	0.01805 (17)	0.0250 (5)	
H9A	0.8830	0.6021	0.0605	0.038*	
H9B	0.8609	0.4775	−0.0386	0.038*	
H9C	0.9339	0.5050	−0.0107	0.038*	
C10	0.76174 (8)	0.4997 (3)	0.26208 (14)	0.0130 (4)	
C11	0.81423 (9)	0.4896 (3)	0.32961 (14)	0.0146 (4)	
H11	0.8524	0.4401	0.3055	0.017*	
C12	0.81130 (9)	0.5511 (3)	0.43132 (15)	0.0153 (4)	
H12	0.8473	0.5421	0.4764	0.018*	
C13	0.75617 (9)	0.6257 (3)	0.46787 (15)	0.0163 (4)	
H13	0.7541	0.6652	0.5381	0.020*	
C14	0.70405 (9)	0.6421 (3)	0.40097 (15)	0.0160 (4)	
H14	0.6663	0.6954	0.4248	0.019*	
C15	0.70724 (8)	0.5805 (3)	0.29941 (14)	0.0123 (4)	
C16	0.59254 (8)	0.5762 (3)	0.24976 (15)	0.0145 (4)	
H16	0.5756	0.5184	0.3091	0.017*	
C17	0.56039 (8)	0.6464 (3)	0.16345 (15)	0.0135 (4)	
C18	0.49267 (9)	0.6540 (3)	0.13925 (15)	0.0151 (4)	
C19	0.46969 (9)	0.7268 (3)	0.04465 (15)	0.0198 (4)	
H19	0.4980	0.7722	−0.0047	0.024*	
C20	0.40557 (9)	0.7336 (3)	0.02211 (16)	0.0240 (5)	
H20	0.3902	0.7846	−0.0422	0.029*	
C21	0.36407 (9)	0.6660 (3)	0.09340 (17)	0.0221 (5)	
H21	0.3203	0.6688	0.0775	0.026*	
C22	0.38639 (9)	0.5945 (3)	0.18778 (17)	0.0223 (4)	
H22	0.3579	0.5493	0.2369	0.027*	
C23	0.45047 (9)	0.5887 (3)	0.21079 (16)	0.0186 (4)	
H23	0.4655	0.5399	0.2758	0.022*	

### Atomic displacement parameters (Å^2^)

**Table:**

	*U*^11^	*U*^22^	*U*^33^	*U*^12^	*U*^13^	*U*^23^
Se	0.01516 (10)	0.02217 (11)	0.01296 (10)	0.00690 (8)	−0.00215 (7)	−0.00303 (8)
N1	0.0107 (7)	0.0140 (8)	0.0142 (8)	0.0002 (6)	0.0017 (6)	0.0018 (7)
N2	0.0137 (8)	0.0231 (9)	0.0157 (8)	0.0002 (7)	0.0015 (6)	0.0047 (7)
N3	0.0119 (8)	0.0213 (9)	0.0182 (8)	0.0012 (7)	0.0001 (6)	0.0031 (7)
C1	0.0109 (9)	0.0163 (9)	0.0121 (9)	0.0018 (7)	−0.0019 (7)	−0.0050 (8)
C2	0.0144 (9)	0.0154 (9)	0.0131 (9)	−0.0029 (8)	0.0009 (7)	−0.0050 (8)
C3	0.0212 (10)	0.0146 (9)	0.0145 (9)	0.0024 (8)	−0.0002 (8)	−0.0005 (8)
C4	0.0155 (10)	0.0244 (11)	0.0141 (9)	0.0035 (8)	−0.0022 (7)	−0.0044 (8)
C5	0.0133 (9)	0.0272 (11)	0.0142 (9)	−0.0034 (8)	0.0013 (7)	−0.0026 (9)
C6	0.0168 (9)	0.0168 (10)	0.0134 (9)	−0.0023 (8)	−0.0002 (7)	−0.0020 (8)
C7	0.0183 (10)	0.0189 (10)	0.0208 (10)	−0.0021 (8)	0.0042 (8)	0.0006 (9)
C8	0.0208 (11)	0.0446 (14)	0.0218 (11)	0.0143 (10)	0.0007 (9)	0.0016 (11)
C9	0.0254 (11)	0.0257 (11)	0.0241 (11)	−0.0018 (9)	0.0027 (9)	0.0078 (10)
C10	0.0162 (9)	0.0105 (9)	0.0124 (9)	−0.0008 (7)	0.0006 (7)	0.0003 (8)
C11	0.0122 (9)	0.0147 (9)	0.0167 (9)	0.0008 (7)	0.0006 (7)	−0.0013 (8)
C12	0.0164 (9)	0.0138 (9)	0.0154 (9)	−0.0005 (8)	−0.0034 (7)	0.0011 (8)
C13	0.0199 (10)	0.0167 (10)	0.0125 (9)	−0.0016 (8)	0.0021 (7)	−0.0013 (8)
C14	0.0134 (9)	0.0154 (9)	0.0196 (10)	0.0012 (7)	0.0052 (8)	−0.0007 (8)
C15	0.0109 (9)	0.0096 (9)	0.0165 (9)	−0.0020 (7)	0.0005 (7)	0.0032 (8)
C16	0.0111 (9)	0.0124 (9)	0.0201 (10)	0.0004 (7)	0.0033 (7)	0.0009 (8)
C17	0.0122 (9)	0.0107 (9)	0.0178 (9)	0.0007 (7)	0.0015 (7)	−0.0014 (8)
C18	0.0137 (9)	0.0116 (9)	0.0199 (10)	0.0010 (7)	0.0005 (8)	−0.0027 (8)
C19	0.0174 (10)	0.0259 (11)	0.0162 (10)	0.0017 (8)	0.0016 (8)	−0.0032 (9)
C20	0.0196 (10)	0.0305 (12)	0.0215 (10)	0.0059 (9)	−0.0055 (8)	−0.0053 (10)
C21	0.0124 (9)	0.0234 (11)	0.0300 (12)	0.0015 (8)	−0.0042 (8)	−0.0089 (10)
C22	0.0145 (10)	0.0182 (10)	0.0344 (12)	−0.0018 (8)	0.0030 (9)	0.0016 (10)
C23	0.0151 (9)	0.0160 (10)	0.0245 (10)	0.0005 (8)	−0.0004 (8)	0.0048 (9)

### Geometric parameters (Å, º)

**Table:**

Se—C10	1.9199 (18)	C9—H9C	0.9800
Se—C1	1.9311 (18)	C10—C15	1.396 (3)
N1—C16	1.347 (2)	C10—C11	1.397 (2)
N1—N2	1.358 (2)	C11—C12	1.385 (3)
N1—C15	1.434 (2)	C11—H11	0.9500
N2—N3	1.311 (2)	C12—C13	1.387 (3)
N3—C17	1.369 (2)	C12—H12	0.9500
C1—C6	1.402 (3)	C13—C14	1.389 (3)
C1—C2	1.403 (3)	C13—H13	0.9500
C2—C3	1.395 (3)	C14—C15	1.384 (3)
C2—C7	1.506 (3)	C14—H14	0.9500
C3—C4	1.393 (3)	C16—C17	1.376 (3)
C3—H3	0.9500	C16—H16	0.9500
C4—C5	1.388 (3)	C17—C18	1.471 (3)
C4—C8	1.504 (3)	C18—C23	1.393 (3)
C5—C6	1.392 (3)	C18—C19	1.394 (3)
C5—H5	0.9500	C19—C20	1.392 (3)
C6—C9	1.510 (3)	C19—H19	0.9500
C7—H7A	0.9800	C20—C21	1.385 (3)
C7—H7B	0.9800	C20—H20	0.9500
C7—H7C	0.9800	C21—C22	1.384 (3)
C8—H8A	0.9800	C21—H21	0.9500
C8—H8B	0.9800	C22—C23	1.392 (3)
C8—H8C	0.9800	C22—H22	0.9500
C9—H9A	0.9800	C23—H23	0.9500
C9—H9B	0.9800		
			
C10—Se—C1	98.26 (8)	C15—C10—C11	117.77 (17)
C16—N1—N2	110.77 (15)	C15—C10—Se	120.88 (13)
C16—N1—C15	129.16 (16)	C11—C10—Se	121.34 (14)
N2—N1—C15	119.66 (14)	C12—C11—C10	120.82 (17)
N3—N2—N1	107.14 (14)	C12—C11—H11	119.6
N2—N3—C17	109.07 (15)	C10—C11—H11	119.6
C6—C1—C2	121.05 (16)	C11—C12—C13	120.54 (17)
C6—C1—Se	118.43 (14)	C11—C12—H12	119.7
C2—C1—Se	120.48 (14)	C13—C12—H12	119.7
C3—C2—C1	118.44 (17)	C12—C13—C14	119.42 (18)
C3—C2—C7	119.49 (17)	C12—C13—H13	120.3
C1—C2—C7	122.06 (17)	C14—C13—H13	120.3
C4—C3—C2	121.86 (18)	C15—C14—C13	119.79 (17)
C4—C3—H3	119.1	C15—C14—H14	120.1
C2—C3—H3	119.1	C13—C14—H14	120.1
C5—C4—C3	118.04 (17)	C14—C15—C10	121.60 (17)
C5—C4—C8	121.29 (19)	C14—C15—N1	118.08 (16)
C3—C4—C8	120.65 (19)	C10—C15—N1	120.30 (16)
C4—C5—C6	122.45 (18)	N1—C16—C17	104.89 (16)
C4—C5—H5	118.8	N1—C16—H16	127.6
C6—C5—H5	118.8	C17—C16—H16	127.6
C5—C6—C1	118.15 (18)	N3—C17—C16	108.13 (16)
C5—C6—C9	119.12 (18)	N3—C17—C18	121.92 (17)
C1—C6—C9	122.73 (17)	C16—C17—C18	129.94 (18)
C2—C7—H7A	109.5	C23—C18—C19	118.95 (17)
C2—C7—H7B	109.5	C23—C18—C17	120.39 (17)
H7A—C7—H7B	109.5	C19—C18—C17	120.66 (18)
C2—C7—H7C	109.5	C20—C19—C18	120.43 (19)
H7A—C7—H7C	109.5	C20—C19—H19	119.8
H7B—C7—H7C	109.5	C18—C19—H19	119.8
C4—C8—H8A	109.5	C21—C20—C19	120.09 (19)
C4—C8—H8B	109.5	C21—C20—H20	120.0
H8A—C8—H8B	109.5	C19—C20—H20	120.0
C4—C8—H8C	109.5	C22—C21—C20	119.93 (18)
H8A—C8—H8C	109.5	C22—C21—H21	120.0
H8B—C8—H8C	109.5	C20—C21—H21	120.0
C6—C9—H9A	109.5	C21—C22—C23	120.1 (2)
C6—C9—H9B	109.5	C21—C22—H22	119.9
H9A—C9—H9B	109.5	C23—C22—H22	120.0
C6—C9—H9C	109.5	C22—C23—C18	120.50 (19)
H9A—C9—H9C	109.5	C22—C23—H23	119.8
H9B—C9—H9C	109.5	C18—C23—H23	119.8
			
C16—N1—N2—N3	−0.6 (2)	C11—C10—C15—C14	−2.5 (3)
C15—N1—N2—N3	−173.99 (15)	Se—C10—C15—C14	177.07 (14)
N1—N2—N3—C17	0.3 (2)	C11—C10—C15—N1	175.45 (16)
C6—C1—C2—C3	0.8 (3)	Se—C10—C15—N1	−4.9 (2)
Se—C1—C2—C3	178.16 (13)	C16—N1—C15—C14	−43.5 (3)
C6—C1—C2—C7	−179.33 (17)	N2—N1—C15—C14	128.46 (18)
Se—C1—C2—C7	−2.0 (2)	C16—N1—C15—C10	138.4 (2)
C1—C2—C3—C4	−0.1 (3)	N2—N1—C15—C10	−49.6 (2)
C7—C2—C3—C4	−179.96 (18)	N2—N1—C16—C17	0.7 (2)
C2—C3—C4—C5	−0.6 (3)	C15—N1—C16—C17	173.24 (17)
C2—C3—C4—C8	177.51 (18)	N2—N3—C17—C16	0.1 (2)
C3—C4—C5—C6	0.7 (3)	N2—N3—C17—C18	179.00 (17)
C8—C4—C5—C6	−177.43 (18)	N1—C16—C17—N3	−0.5 (2)
C4—C5—C6—C1	0.0 (3)	N1—C16—C17—C18	−179.26 (18)
C4—C5—C6—C9	179.89 (18)	N3—C17—C18—C23	−176.52 (18)
C2—C1—C6—C5	−0.8 (3)	C16—C17—C18—C23	2.1 (3)
Se—C1—C6—C5	−178.15 (14)	N3—C17—C18—C19	3.3 (3)
C2—C1—C6—C9	179.32 (18)	C16—C17—C18—C19	−178.05 (19)
Se—C1—C6—C9	1.9 (2)	C23—C18—C19—C20	−0.2 (3)
C15—C10—C11—C12	2.5 (3)	C17—C18—C19—C20	179.96 (18)
Se—C10—C11—C12	−177.11 (14)	C18—C19—C20—C21	−0.6 (3)
C10—C11—C12—C13	−0.6 (3)	C19—C20—C21—C22	1.0 (3)
C11—C12—C13—C14	−1.3 (3)	C20—C21—C22—C23	−0.6 (3)
C12—C13—C14—C15	1.3 (3)	C21—C22—C23—C18	−0.2 (3)
C13—C14—C15—C10	0.7 (3)	C19—C18—C23—C22	0.6 (3)
C13—C14—C15—N1	−177.35 (16)	C17—C18—C23—C22	−179.55 (18)

### Hydrogen-bond geometry (Å, º)

Cg1, Cg2 and Cg3 are the centroids of the C1–C6, C10–C15 and C18–C23 rings, respectively.

**Table:**

*D*—H···*A*	*D*—H	H···*A*	*D*···*A*	*D*—H···*A*
C12—H12···*Cg*1^i^	0.95	2.68	3.481 (2)	143
C7—H7a···*Cg*2^ii^	0.98	2.61	3.492 (2)	150
C16—H16···*Cg*3^iii^	0.95	2.66	3.399 (2)	135

Symmetry codes: (i) *x*, −*y*−1/2, *z*−1/2; (ii) *x*, *y*−1, *z*; (iii) −*x*+1, *y*−1/2, −*z*+1/2.
