# Supplementary figures and images for: Crystal structure of 4-phenyl-1-{2-[(2,4,6-tri­methyl­phen­yl)selan­yl]phen­yl}-1H-1,2,3-triazole
Source: Acta Crystallogr E Crystallogr Commun. 2015 Feb 25;71(Pt 3):o204–5. doi: 10.1107/S2056989015003229 (PMC4350715; doi:10.1107/S2056989015003229)

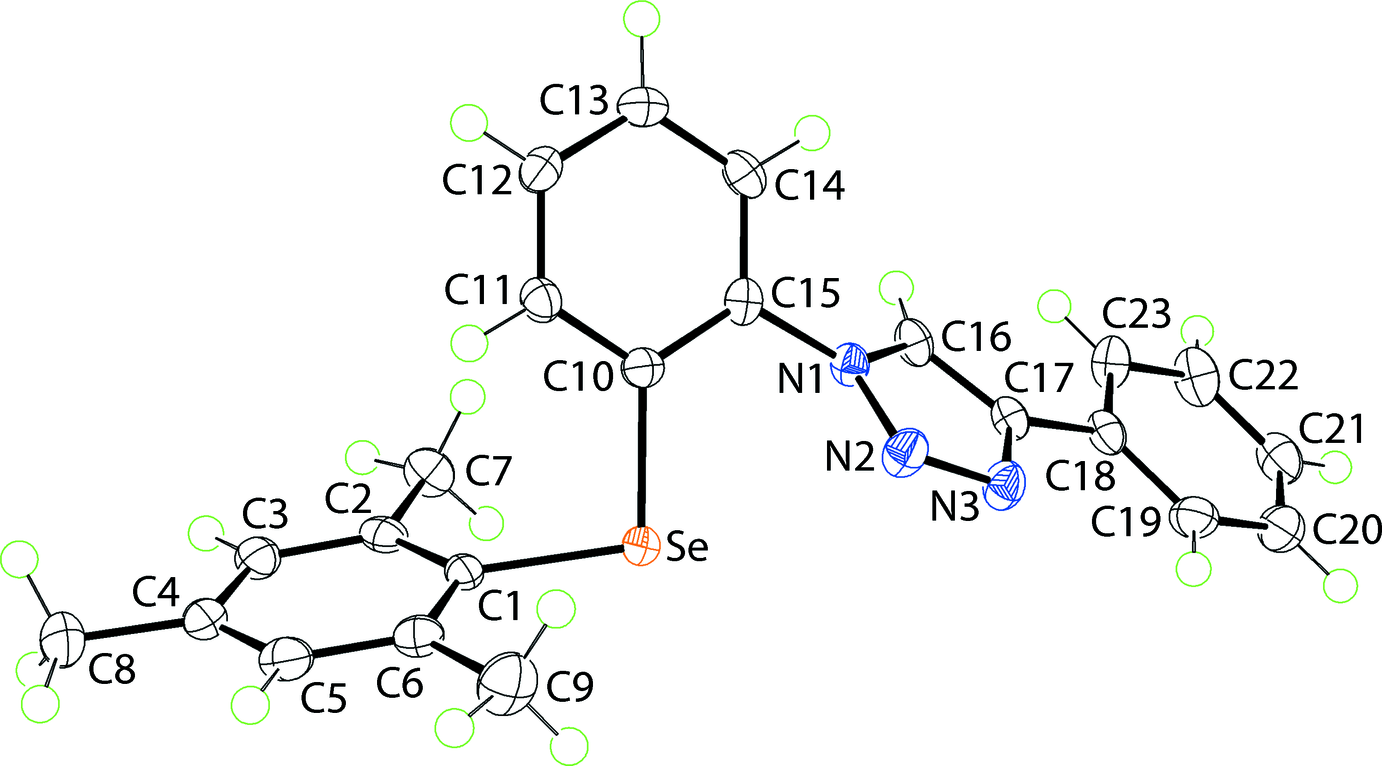

Supplement: Supplementary file 4 [file e-71-0o204-fig1.tif]

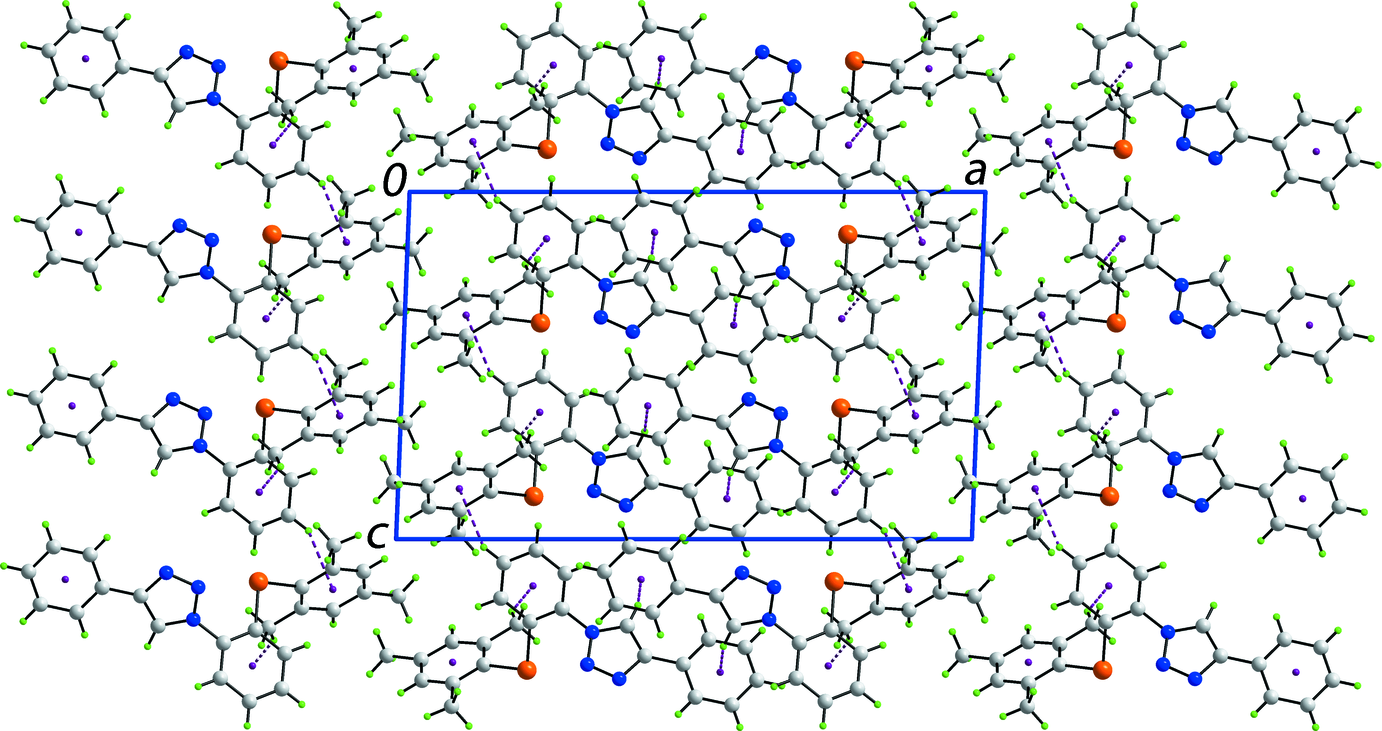

Supplement: Supplementary file 5 [file e-71-0o204-fig2.tif]
